# Supplementary material for: Prevalence and predictors of anemia among adults on highly active antiretroviral therapy in Northeast Ethiopia: A retrospective cohort study
Source: PLoS One. 2022 Mar 25;17(3):e0265337. doi: 10.1371/journal.pone.0265337 (PMC8956168; doi:10.1371/journal.pone.0265337)
Supplement: Attachment — Submitted filename: Comments to PLOS One.docx [file pone.0265337.s002.docx]

Reviewer’s Comments

***Prevalence and predictors of anemia among adults on highly active***

***antiretroviral therapy in Northeast Ethiopia: a retrospective cohort study***

The duration of the study is between September 2005 to January 2018 in the abstract but its September 2005 to January 2019 in the methodology. Which is which?

It’s quite difficult to understand when the hemoglobin measurement was taken. The paper does not clearly state the timelines for hemoglobin measurements.

The stratification of the patients is not clear. The authors need to clearly define how their subjects were selected; what parameter was measured at what time. Was there baseline measurements and subsequent measurements of hemoglobin? Were all the subjects on ART at certain point? What was the point of including the naïve subjects when the work was clearly looking at effect of HAART on hemoglobin levels?

Anemia is multifactorial in nature. How were the authors able to control for other confounders which could affect the level of hemoglobin?

Why was the TDF based ART regimen associated with anemia in the study? No reason was assigned to this in the discussion.

Why was there no bivariate analysis between duration of HAART and the risk of developing anemia?
