# Supplementary material for: Prevalence and predictors of anemia among adults on highly active antiretroviral therapy in Northeast Ethiopia: A retrospective cohort study
Source: PLoS One. 2022 Mar 25;17(3):e0265337. doi: 10.1371/journal.pone.0265337 (PMC8956168; doi:10.1371/journal.pone.0265337)
Supplement: Attachment — Submitted filename: Reviewer Report.docx [file pone.0265337.s003.docx]

**Reviewer Report to Editor (s)**

**Prevalence and predictors of anemia among adults on highly active antiretroviral therapy in Northeast Ethiopia: a retrospective cohort study (PONE-D-21-25369)**

Dear Editor, first, I would like to thank for your invitation to review the above MS. This MS might give important information about **Prevalence and predictors of anemia among adults on highly active antiretroviral therapy** for clinicians and the MS is almost well written. But, before it is going to be considered for publication, the authors need to address some of the concerns indicated below. In addition, the MS needs language proof reading. There are many topographic errors. The MS need to be accepted after revision.

**Reviewer Report to the author (s)**

This MS might give important information about **Prevalence and predictors of anemia among adults on highly active antiretroviral therapy** for clinicians and for the scientific community. But the authors need to address the following points before publication:

- The authors name in the cover page can be rewrite as “Teklehaimanot Fentie Wendie1*, Kassahun Bogale1, Getnet Mengistu1”
- The corresponding author Email address in the cover page can be modifies as :

E-mail: [tklhmntfnt5@gmail.com(TF)](mailto:tklhmntfnt5@gmail.com(TF)) ([Teklehaimanot.fentie@wu.edu.et](mailto:Teklehaimanot.fentie@wu.edu.et))

- In the abstract, method subsection, the authors need to indicate the specific study sites at the end of the study period: A retrospective cohort study was carried out among adults who began highly active antiretroviral treatment between September 2005 and January 2018 in ----------.
- From the abstract, the authors need to avoid the use of abbreviations (Eg. HARRT, ART, TDF, etc) before indicating their full names.
- In this MS, there is unnecessary excessive use of references throughout the document after single sentences. The authors need to be specific and better to use only recent and relevant one or two references after a sentence of paragraphs.
- The introduction part needs some rearrangements for simplicity for readers. The authors first indicate the global burden of anemia on HIV positive individuals, then in African and Ethiopia and the study area particularly.
- The title or the subtitles may not be necessarily indicated on separate pages. For example, the methodology part need to be indicated immediately after introduction part in the same page.
- The title “Method and participants” can be modified as “Methodology” or “Methods and Materials”. Based on this modification, the subtitle “methods” in the abstract section can be modified.
- In your methodology part, the study area is not well elaborated. In addition, the authors didn’t clearly indicate the reason why only two government hospitals were selected among the others.
- Since your title focuses to see the condition of anemia among HIV positive individuals who are on HAART, it is not clear the reason why you include study participants started from the age of 15 years. In Ethiopian context, the word “adult” refers individuals’ grater or equal to 18 years. Please reconsider it.
- Based on your sample size assumptions, the calculated sample size will be around 383(4). But as indicated in your result, the final sample size was 392. It needs justification.
- The hemoglobin level was evaluated after adjusting the attitude and smoking status. But it is not clearly indicated in the methods part. It seems theoretical assumption.
- It is better to indicate where the pretest of the data extraction tool was tested. It is not clearly indicated.
- In your ‘ethical consideration” subtitle, the phrase “….in compliance with the Helsinki Declaration on the ethical principles of medical research involving human subject…” is not important. Better to be deleted.
- Since your title is among adults on highly active antiretroviral therapy, how 233 (59.4%) of them were naïve to ART in your result? It is very confusing.
- In result at the time of enrollment, what was the prevalence of anemia? It is not indicated. But is very important for comparison to see the prevalence of anemia before and after the initiation of ART.
- It is better to insert the respective tables immediately after the text it refers.
- Indicating the number of study participants (N=392) at the end of the titles of the table is not convincing for the reader; it is better to be deleted.
- Since the authors already indicated the meaning of “COR” and “AOR” in “Predictors of anemia” subtitle, no need of indicating them at the end of table 3 as key word. This concept can also hold true for the other abbreviations indicated in the respective tables.
- In your discussion part, second paragraph, the phrase “….defined as Hgb concentration <13 g/dl for males and <12 g/dl for females after adjustment for altitude and smoking status of measured values…” is not important. It is almost reputation. Better to be deleted.
- When you compare results in discussion, you need to have 95% CI in your result. For example the prevalence of anemia in your result is 41.3%. But you didn’t indicate its 95%CI. Hence it is very difficult to say comparable, lower or higher than other reports. This can be true for other variables that you compare.
- From your conclusion, avoid unnecessary explanations. For example avoid “(100 - 200 cells/mm3)”
